# Formation of N^ε^-Carboxymethyl-Lysine and N^ε^-Carboxyethyl-Lysine in Heated Fish Myofibrillar Proteins with Glucose: Relationship with Its Protein Structural Characterization

**DOI:** 10.3390/foods12051039

**Published:** 2023-03-01

**Authors:** Siqi Zhang, Pengcheng Zhou, Peng Han, Hao Zhang, Shiyuan Dong, Mingyong Zeng

**Affiliations:** College of Food Science and Engineering, Ocean University of China, Qingdao 266003, China

**Keywords:** advanced glycation end products, thermal treatment, myofibrillar proteins, aggregation, N^ε^-carboxymethyl-lysine, N^ε^-carboxyethyl-lysine

## Abstract

The formation of advanced glycation end products (AGEs), including N^ε^-carboxymethyl-lysine (CML) and N^ε^-carboxyethyl-lysine (CEL), in a fish myofibrillar protein and glucose (MPG) model system at 80 °C and 98 °C for up to 45 min of heating were investigated. The characterization of protein structures, including their particle size, ζ-potential, total sulfhydryl (T-SH), surface hydrophobicity (H_0_), sodium dodecyl sulfate–polyacrylamide gel electrophoresis (SDS–PAGE) and Fourier transform infrared spectroscopy (FTIR), were also analyzed. It was found that the covalent binding of glucose and myofibrillar protein at 98 °C promoted protein aggregation when compared with the fish myofibrillar protein (MP) heated alone, and this aggregation was associated with the formation of disulfide bonds between myofibrillar proteins. Furthermore, the rapid increase of CEL level with the initial heating at 98 °C was related to the unfolding of fish myofibrillar protein caused by thermal treatment. Finally, correlation analysis indicated that the formation of CEL and CML had a significantly negative correlation with T-SH content (r = −0.68 and r = −0.86, *p* ≤ 0.011) and particle size (r = −0.87 and r = −0.67, *p* ≤ 0.012), but was weakly correlated with α-Helix, β-Sheet and H_0_ (r^2^ ≤ 0.28, *p* > 0.05) during thermal treatment. Overall, these findings provide new insights into the formation of AGEs in fish products based on changes of protein structure.

## 1. Introduction

The Maillard reaction is one of the common reactions during food thermal processing, which plays a vital role in the formation of food flavor and color [1,2,3]. Advanced glycation end products (AGEs) are chemicals formed at the advanced stage of the Maillard reaction and regarded as harmful substances [4]. It is worth noting that long-term exposure to a high AGEs diet could cause accumulation in the body and promote inflammation, which may cause some chronic diseases such as diabetes and atherosclerosis [5,6]. Some AGEs, especially lysine-derived N^ε^-carboxymethyl-lysine (CML) and N^ε^-carboxyethyl-lysine (CEL), are most abundant and stable in protein and fat-rich foods, such as meat and meat products [1,7], and widely studied in foods [8,9,10].

The formation of CEL and CML in cooked fish products depends on the food composition and the type of thermal treatment, heating temperature and time. Niu et al. (2017) [11] reported that boiling at 100 °C for 30 min resulted in a significant increase in protein-bound CML (2.1–10.8-fold increase) and CEL (27–242% increase) from grass carp muscle. Liu et al. (2022) [12] showed that production of fluorescent AGEs in sturgeon fillets was greatly increased by frying time, and both frying temperature and time had an extremely significant effect on CML and CEL levels (*p* < 0.001). Our previous study showed that CML levels in fried hairtail fillets were higher than in boiled and baked ones, regardless of the cooking time [10].

Notably, since proteins are macromolecules with complex advanced structures, the glycation of protein during thermal treatments can also be regulated by changing the structure of the protein [13]. Recently, the interaction between the changes of protein structure and its glycation extent during thermal processing treatments has attracted extensive attention. By analyzing proteomics, Xu et al. (2020) [14] showed that ultrasonic pretreatment (UP) at 20 kHz induced in protein unfolding and aggregation behavior in a bovine serum albumin (BSA) or β-lactoglobulin (β-Lg) model system, which changed the glycation extent of the Lys and Arg. In addition, Huang et al. (2013) [15] monitored the number of glycation sites of ovalbumin by Fourier transform ion cyclotron mass spectrometry (FTICR-MS) before and after reducing the pair of the intrachain disulfide bond, and found that when the ovalbumin tertiary structure was disrupted after reducing the disulfide bond, the number of glycated sites of the protein increased. It is worth noting that mild oxidation led to the unfolding of pork myofibrillar protein and exposed more free amino acids, which facilitated the formation of CML by the Maillard reaction [16]. However, few studies have examined the relationship between the structure changes of fish protein and the formation of CML and CEL via glycation reaction.

In recent years, sturgeon aquaculture has developed rapidly in China, with an estimated annual harvest of 121,875 metric tons in 2021 (China Fishery Statistical Yearbook, 2022). Sturgeon is a precious food source with many active components that are beneficial for human’s health, and has high nutritional and economic value worldwide [17]. Thermal processing methods, such as steaming, boiling, baking or frying have been applied to treat sturgeon meat products [18,19]. Myofibrillar protein, accounting for 55–65% of muscle proteins, is the predominant component of proteins in fish muscle [20]. Our hypothesis is that the structural changes of myofibrillar protein (MP) during thermal treatments may affect the formation process of AGEs. Therefore, the structure properties of myofibrillar protein and glucose during the heating process were determined by total sulfhydryl, surface hydrophobicity, free amino content, particle size, ζ-potential, Fourier transform infrared spectroscopy, sodium dodecyl sulfate-polyacrylamide gel electrophoresis, and the formation of corresponding furosine, N^ε^-carboxymethyl-lysine (CML) and N^ε^-carboxyethyl-lysine (CEL) were analyzed. The correlations between these parameters and AGEs were also analyzed. These findings provide a theoretical basis for revealing the mechanism of AGEs formation in fish products during cooking or processing treatments.

## 2. Materials and Methods

### 2.1. Chemicals

N^ε^-carboxymethyl-lysine (CML), N^ε^-carboxyethyl-lysine (CEL), N^ε^-carboxymethyl-lysine-d4 (CML-d4), and N^ε^-carboxyethyl-lysine-d4 (CEL-d4) were bought from Toronto Research Chemicals Inc. (Toronto, Canada). Acetonitrile of HPLC grade was purchased from Merck (Darmstadt, Germany). All the other chemicals and reagents used in this study were of analytical grade.

### 2.2. Sample Preparation

Fresh hybrid sturgeons (*Acipenser baerii × Acipenser schrenckii)*, weighing 1.8 ± 0.2 kg and 65 ± 5 cm in length, were purchased from a local sturgeon farm in Qingdao (Shandong province, China) and transported to the laboratory within an hour. The head, bones, and skin of the fish were manually removed and the fresh flesh was used for myofibrillar protein (MP) extraction.

The MP was extracted according to the method of Han et al., (2017) [21] with some modifications. The protein concentration was evaluated by the Biuret method [22].

For myofibrillar protein and glucose (MPG) heated samples, MP (10 mg mL^−1^) and glucose at the ratio of 10:1 (*w/w*) were dissolved in 50 mM phosphate buffer solution (pH 7.0). The solution was kept in 25 mL screw-cap tubes and heated in a water bath at 80 °C and 98 °C for 2.5, 5, 10, 15, 30 and 45 min, respectively. The choice of heating condition is according to light (80 °C) or strong (98 °C) cooking or processing of some fish foods [11,19]. Control experiments with MP suspensions (10 mg mL^−1^) heated without glucose were also conducted. The MP and MPG samples were stored at −60 °C for further analysis.

### 2.3. Analysis of CML and CEL

The levels of CML and CEL were assessed by the method of Sun et al. (2015) [23] with minor modifications. Briefly, two milliliters of sample suspension (5 mg mL^−1^) were incubated with 0.4 mL borate buffer (0.2 M, pH 9.2) and 0.08 mL sodium borohydride (2 M, dissolved with 0.1 M NaOH) at 4 °C for 8 h and then hydrolyzed by 1.6 mL 6 M HCl at 110 °C for 24 h. Next, the protein hydrolysate was dried in a vacuum oven (DZF-6050; Shanghai Jinghong Laboratory instrument Co., Ltd., Shanghai, China) at 60 °C and diluted with water to 4 mL, from which 1 mL was withdrawn and spiked with 20 μL internal standard (CML-d4, CEL-d4). After activating and balancing a Sep-Pak MCX column, the sample was purified by this column and eluted with 2 mL methanol containing 5% ammonia water. Finally, the eluent was dried in nitrogen with a nitrogen evaporator (DC12H; Shanghai ANPEL Scientific Instrument Co., Ltd., Shanghai, China), reconstituted with 2 mL deionized water, and filtered through a 0.22 µm filter before LC-MS/MS analysis.

### 2.4. Analysis of Furosine

Furosine was determined according to the method described by Semedo Tavares et al. (2018) [10] with a slight modification. The analyses were performed using a HPLC system (Agilent 1100, San Leandro, CA, USA) equipped with an Alltima C8 column (250 mm × 4.6 mm, 5 μm, Grace Davison, Columbia, MD, USA). The column (250 × 4.6 mm Alltech, Nicholasville, KY, USA) temperature was set at 30 °C and the UV/VIS detector set at 280 nm. The mobile phase was performed at a flow rate of 1 mL/min with (A) water containing 0.4% acetic acid, and (B) 0.3% potassium chloride (KCl) in (A). The mobile phase consists of 98% liquid A and 2% liquid B.

### 2.5. Total Sulfhydryl (T-SH)

The T-SH content of samples was determined according to the method of Ellman (1959) [24]. The results were calculated by using a molar extinction coefficient of 1.36 × 104 M/cm and expressed in micromoles of T-SH per gram of protein.

### 2.6. Protein Surface Hydrophobicity (H_0_)

Bromophenol blue (BPB) can adhere the surface hydrophobic region of soluble proteins and insoluble proteins and is therefore used to estimate protein surface hydrophobicity [25,26]. The H_0_ content of samples was measured by the method of Zhang et al. (2020) [27] with some modifications. A total of 40 μL 1 mg mL^−1^ BPB (in deionized water) was added to 2 mL samples and the solution was stirred at 200 rpm for 10 min at room temperature. The mixtures were centrifuged for 10 min at 2000× *g*, and then the absorbance of the supernatant was measured at 595 nm. The results were expressed as the content of bound BPB.

### 2.7. Sodium Dodecyl Sulfate–Polyacrylamide Gel Electrophoresis (SDS-PAGE)

The SDS–PAGE was performed using the method of Zhou et al. (2021) [28] with an 10% separating gel and a 5% stacking gel. Coomassie Brilliant Blue R was used for protein staining.

### 2.8. Fourier Transform Infrared Spectroscopy (FTIR)

The FTIR was determined according to the method described by Ren et al. (2022) [29] with some modifications. A Fourier transform spectrophotometer (Nicolet iS10, Thermo Scientific Corp., Madison, WI, USA) was used to obtain the infrared absorption value of samples. The dried powder samples were diluted with spectral grade potassium bromide (KBr) and the absorbance spectrum of KBr as background was used to eliminate interference for samples. The scanning range was 4000–400 cm^−1^ with a resolution of 4 cm^−1^ and 64 scans for each sample.

### 2.9. Particle Size and ζ-Potential

The average particle size and ζ-potential of samples were determined by using a laser particle size analyzer (Malvern Nano ZS, Malvern Instruments Ltd., Malvern, Worcestershire, UK) at 25 °C. Samples were diluted approximately 4-fold with the same buffer, mixed, and immediately transferred into plastic cuvettes for determination.

### 2.10. Free Amino Content

The free amino content of samples was quantified by the OPA (o-phthalaldehyde) spectrophotometric assay, as described by Church et al., (1983) [30]. The OPA reagent was prepared daily by combining the following reagents and diluting to a final volume of 50 mL with distilled water: 40 mg of OPA (dissolved in 1 mL of methanol), 25 mL of 100 mM sodium tetraborate, 2.5 mL of 20% (*w/w*) SDS and 100 μL of β-mercaptoethanol. One hundred microliters of the solution of samples (5 mg mL^−1^) was added directly to 2 mL of OPA reagent and left at 35 °C in the dark for 2 min. The absorbance was measured at 340 nm. The standard curve was prepared with lysine at the range of 0–3 mmol L^−1^.

### 2.11. Statistical Analysis

All experiments were performed with three repeats (n = 3) and data expressed as mean ± standard deviation (SD). The statistical analysis was performed using SPSS 25 software. Significant differences between samples were identified at *p* < 0.05 by multi-way analysis of variance (ANOVA). Pearson’s correlation test evaluated the correlation between AGEs formation and structure changes of protein, and *p* < 0.05, *p* < 0.01 and *p* < 0.001, respectively, represented different levels of statistical significance.

## 3. Results and Discussion

### 3.1. CML and CEL Levels

**Figure 1:**
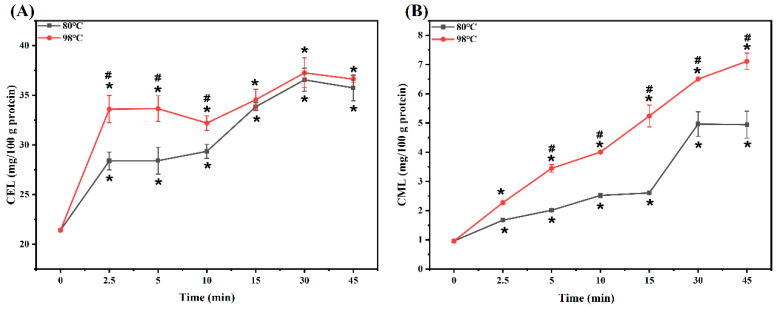
Changes in N^ε^-carboxyethyl-lysine (CEL) (**A**) and N^ε^-carboxymethyl-lysine (CML) (**B**) content of myofibrillar protein and glucose (MPG) under different heating conditions (80 °C and 98 °C, 0–45 min). Error bars indicate standard deviation (n = 3). Samples with * *p* < 0.05 indicate significant differences at different heating times (compared with 0 min). Samples with ^#^ *p* < 0.05 indicate significant differences between 80 °C and 98 °C.

The CEL and CML levels of MPG under different heating conditions are shown in Figure 1A,B, respectively. At both temperatures, the CML level of MPG significantly increased with heating time. Interestingly, the CEL level of MPG within the first 2.5 min of heating at 98 °C dramatically increased by 56.94% when compared with the unheated samples, but then slightly increased by 10.88% from 2.5 min to 30 min. At 80 °C, the CEL level in MPG sharply increased by approximately 33% within the first 2.5 min of heating when compared with the unheated samples, but only increased by 3.43% from 2.5 min to 10 min, and then significantly increased by 24.51% from 10 min to 30 min. Moreover, within the first 10 min of heating, the CEL level of MPG at 98 °C was much higher than that at 80 °C, but there was no significant difference of CEL level between these two temperatures for 15 min to 45 min of heating. On the other hand, the CEL level of MPG during the whole heating process was much higher than that of CML, suggesting that myofibrillar protein and glucose were more likely to produce CEL under such thermal treatment conditions. Our previous study also found that the CEL level in fried sturgeon fillets was much higher than that of the CML [12].

To the best of our knowledge, the reason for the sharp increase of the CEL level during the initial heating stage is not clear. Yu et al. (2018) [16] found that, when the myofibrillar protein–glucose–linoleic acid system was mildly oxidized during heating, the myofibrillar protein began to unfold, and the exposed free amino groups could facilitate AGEs generation by the Maillard reaction pathway. Xu et al. (2020) [14] reported that five cycles of ultrasonic pretreatment (UP) up-regulated the glycation degree of BSA and β-Lg, possibly due to the unfolding behavior of protein induced by UP, which exposed additional glycation. Thus, we speculated that, during the initial heating, the rapid increase of CEL might be related to the unfolding of fish myofibrillar protein caused by thermal treatment. However, our results demonstrated that the formation of CEL was not obviously affected by further heating at higher temperatures, which might be attributed to the aggregation of fish myofibrillar protein caused by deeper thermal treatments to hide some glycation sites [13].

### 3.2. Furosine

**Figure 2:**
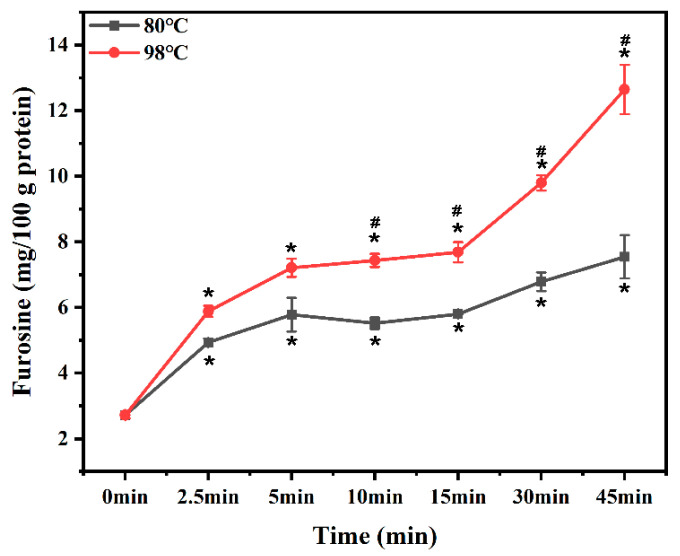
Changes in furosine content of myofibrillar protein and glucose (MPG) under different heating conditions (80 °C and 98 °C, 0–45 min). Error bars indicate standard deviation (n = 3). Samples with * *p* < 0.05 indicate significant differences at different heating times (compared with 0 min). Samples with ^#^ *p* < 0.05 indicate significant differences between 80 °C and 98 °C.

Furosine is an important indirect indicator of Amadori products and often related to early stage Mailard reaction products [31]. Regardless of temperatures, the furosine content in MPG during the first 2.5 min of heating was markedly increased, but there were no significant changes during 5 min to 15 min of heating (Figure 2). A reasonable explanation might be that, in the later stage of heating, even though the Maillard reaction of fish myofibrillar protein and glucose produced a lot of furosine, some of these compounds could become degraded as Maillard progressed, generating intermediate and end products [32]. Furthermore, when MPG was heated at 80 °C and 98 °C for 45 min, the furosine increases were 30.09% and 64.55%, respectively, more than those after 15 min. This was a similar trend to that observed by Mitra et al., (2018) [33], who reported that no significant changes in furosine content from pork samples heated at 58 °C for 72 min were found, but a longer heating time (17 h) significantly increased furosine content.

### 3.3. Total Sulfhydryl Content

**Figure 3:**
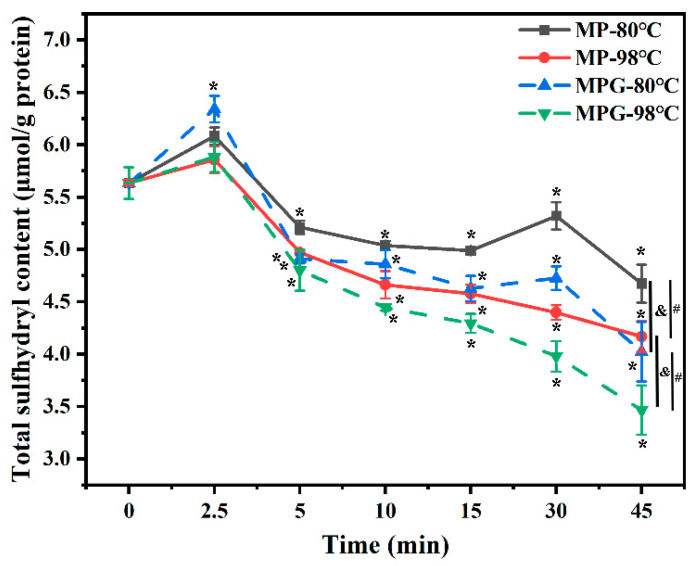
Changes in total sulfhydryl content of myofibrillar protein and glucose (MPG) and myofibrillar protein (MP) under different heating conditions (80 °C and 98 °C, 0–45 min). Error bars indicate standard deviation (n = 3). Samples with * *p* < 0.05 indicate significant differences at different heating times (compared with 0 min). Samples with ^#^ *p* < 0.05 indicate significant differences between 80 °C and 98 °C. Samples with ^&^ *p* < 0.05 indicate significant differences between MPG and MP.

The total sulfhydryl (T-SH) content of MP and MPG greatly varied with heating time and temperature (Figure 3). The T-SH content of MPG within the first 2.5 min of heating rapidly increased, and then significantly decreased with further heating. During the whole heating process, the T-SH content of MPG heated at 98 °C was much lower than that at 80 °C. A decrease in T-SH content was reported to be due to the fact that the sulfhydryl groups of protein intra-molecules formed disulfide bonds during heating [22]. Jiménez-Castaño et al. (2005) [34] documented that dry heating β-Lg with dextran enhanced polymerization of protein, and this polymerization occurred due to disulfide bonds. In this study, when heated at the same temperature for 10 min to 45 min, the T-SH content of MPG was much lower than that of MP. We surmised that this phenomenon was due to the fact that fish myofibrillar protein heated in the presence of glucose promoted protein aggregation to some degree, which caused the sulfhydryl groups of myofibrillar protein to form disulfide bonds.

### 3.4. Surface Hydrophobicity Content

**Figure 4:**
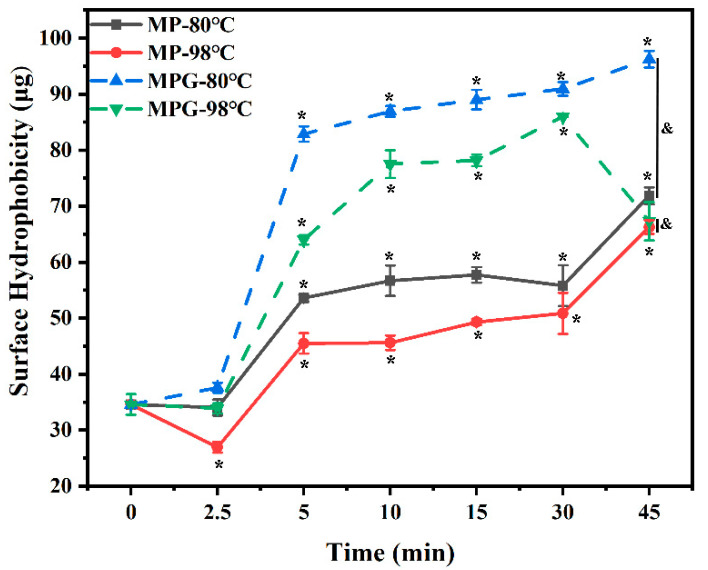
Changes in surface hydrophobicity of myofibrillar protein and glucose (MPG) and myofibrillar protein (MP) under different heating conditions (80 °C and 98 °C, 0–45 min). Error bars indicate standard deviation (n = 3). Samples with * *p* < 0.05 indicate significant differences at different heating times (compared with 0 min). Samples with ^&^ *p* < 0.05 indicate significant differences between MPG and MP.

Analysis of the surface hydrophobicity (H_0_) of molecules can be used to reflect the change of protein conformation [35]. The H_0_ of MPG and MP under different heating conditions is shown in Figure 4. Compared with the unheated samples, the H_0_ content of MPG and MP after heating greatly increased. Moreover, the H_0_ of MPG during the whole process was much higher than that of MP except after 2.5 min of heating. To the best of our knowledge, reasons for the changes of surface hydrophobicity in heated food protein and carbohydrates remain controversial. Jiang et al. (2021) [36] observed that the decrease of surface hydrophobicity of α-lactalbumin heated with xylose was possibly due to the protection of the surface hydrophobic groups by the attached xylose molecules when compared with α-lactalbumin heated alone. However, our results are similar to those of Bian et al. (2018) [37], who reported that the chicken myofibrillar protein heated with glucosamine resulted in higher surface hydrophobicity than myofibrillar protein heated alone, which might be related to protein unfolding. In our present study, the surface hydrophobicity of fish myofibrillar proteins in the presence of glucose was higher than that of fish myofibrillar proteins alone during heating, probably due to aggregation dissociation or protein unfolding [38].

### 3.5. SDS–PAGE

**Figure 5:**
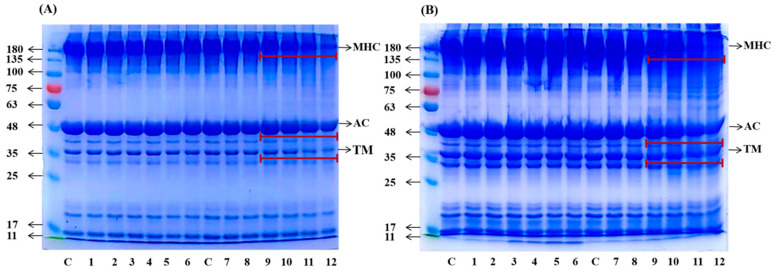
Sodium dodecyl sulfate–polyacrylamide gel electrophoresis profiles of myofibrillar protein (MP) (**A**) and myofibrillar protein and glucose (MPG) (**B**) under different heating conditions (80 °C and 98 °C, 0–45 min). Lanes: c = 0 min; 1 = 80 °C, 2.5 min; 2 = 80 °C, 5 min; 3 = 80 °C, 10 min; 4 = 80 °C, 15 min; 5 = 80 °C, 30 min; 6 = 80 °C, 45 min; 7 = 98 °C, 2.5 min; 8 = 98 °C, 5 min; 9 = 98 °C, 10 min; 10 = 98 °C, 15 min; 11 = 98 °C, 30 min; 12 = 98 °C, 45 min; MHC: myosin heavy chain; AC: actin chain; TM: tropomyosin chain.

The electrophoretic pattern of MP (Figure 5A) and MPG (Figure 5B) under different heating conditions was monitored by SDS–PAGE. The myosin heavy chain (MHC), actin chain (AC) and tropomyosin chain (TM) bands intensity of MPG and MP heated at 80 °C did not decrease with heating time (channels 1 to 6). When MP was heated at 98 °C for 15 min to 45 min, the MHC, AC and TM bands intensity markedly decreased with heating time and. after 45 min of heating, the MHC band almost disappeared (channels 10 to 12), indicating self-aggregation of MP [22]. However, no obvious decrease in MHC, AC and TM bands intensity of MPG were observed at 98 °C for 10 min to 45 min, which could be attributed to the fact that steric hindrance due to glucose conjugated on the MP contributed to the decrease of MP self-aggregation propensity during heating [39]. In addition, the band above 180 KDa of MPG at 98 °C during the whole heating treatment was much denser than that at 80 °C. It is worth noting that, regardless of heating temperature, the band above 180 KDa of MPG was denser than that of MP during the whole heating process, suggesting that heating caused the covalent link of fish myofibrillar protein with glucose to form larger molecular mass polymers than myofibrillar proteins heated alone [40]. The protein polymers in this large molecular mass could be from myosin heavy chain, actin and tropomyosin, as the bands intensity of these proteins were reduced or missing during heating. This finding was consistent with that reported by Bian et al. (2018) [37], who revealed that myosin heavy chain, actin chain, tropomyosin chain or myosin light chain were the main protein reactants in the glycation of chicken myofibrillar protein heated with glucosamine. The present results further confirmed that fish myofibrillar protein and glucose heated at a higher temperature and for a longer heating time promoted protein aggregation, when compared with fish myofibrillar protein heated alone.

### 3.6. FTIR Analysis

**Figure 6:**
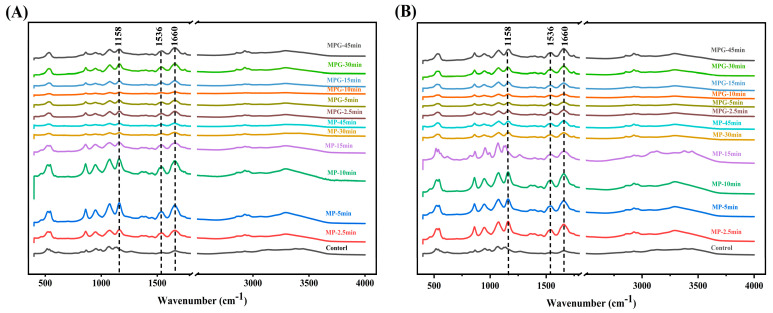
Changes in Fourier transform infrared spectroscopy of myofibrillar protein and glucose (MPG) and myofibrillar protein (MP) heated at 80 °C (**A**) and 98 °C (**B**) for 0–45 min.

The bands at 1600–1700 cm^−1^ and 1450–1550 cm^−1^ from amide I and II groups referred to C=O and C–N stretching, respectively [41]. As reported by Gu et al. (2010) [42], changes in the bands at 1180–953 cm^−1^ could correspond to the stretching of C–C and C–O and the bending mode of C–H bonds. Figure 6A,B show that, in our present study, the absorption intensities of MPG at wavenumbers of 1660 cm^−1^ (amide I), 1536 cm^−1^ (amide II), and 1158 cm^−1^ were much lower than those of MP at the same heating temperature. Qu et al. (2018) [35] indicated that functional groups such as -NH_2_ in proteins, especially lysine, were reduced and the absorption intensity at the wavenumbers of 1650–1600 cm^−1^, 1600–1500 cm^−1^, and 1200–1300 cm^−1^ decreased with the Maillard reaction of rapeseed protein isolate and dextran. Our previous study also found that decreased absorption intensity around the wavenumbers of 1680 cm^−1^, 1540 cm^−1^, and 1153 cm^−1^ were associated with covalent binding of silver carp myofibrillar protein to glucose during heating [29].

**Figure 7:**
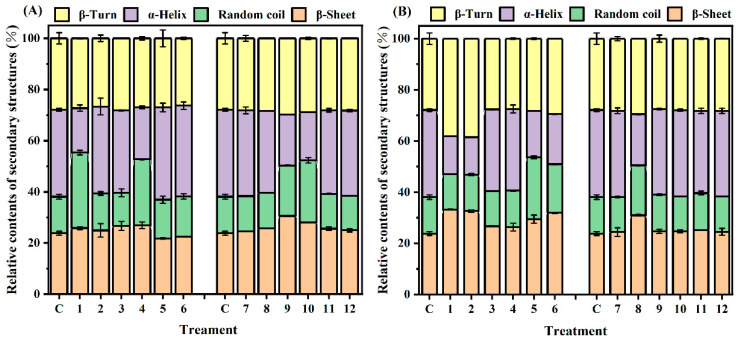
Changes in relative contents of secondary structures of myofibrillar protein and glucose (MPG) and myofibrillar protein (MP) heated at 80 °C (**A**) and 98 °C (**B**) for 0–45 min. c = 0 min; 1 = MP, 2.5 min; 2 = MP, 5 min; 3 = MP, 10 min; 4 = MP, 15 min; 5 = MP, 30 min; 6 = MP, 45 min; 7 = MPG, 2.5 min; 8 = MPG, 5 min; 9 = MPG, 10 min; 10 = MPG, 15 min; 11 = MPG, 30 min; 12 = MPG, 45 min.

Further information about secondary structure contents of MP and MPG under different heating conditions was calculated by PeakFit 4.12 software and are shown in Figure 7. The α-helix content of MPG heated at 80 °C for 10 min to 45 min was markedly lower than that of MP, and the corresponding β-sheet content was significantly higher than that of MP; at 98 °C, the opposite trend was observed. Typically, α-helix structures are buried in the interior sites of polypeptide chains and related to stability of protein [43]. In this sense, evaluation of the secondary structure showed that the binding of glucose and fish myofibrillar protein during heating at 80 °C caused fish myofibrillar protein to become more flexible and disordered [44]. However, heating with a higher temperature (98 °C) might cause more glucose to bind to myofibrillar proteins, and the introduction of more hydroxyl groups from glucose causes an intermolecular interaction among the neighboring proteins, thereby increasing the α-helix content [39]. From the present results, the covalent binding of glucose and fish myofibrillar protein at a higher heating temperature could affect the secondary structure of myofibrillar protein.

### 3.7. Particle Size and ζ-Potential

**Figure 8:**
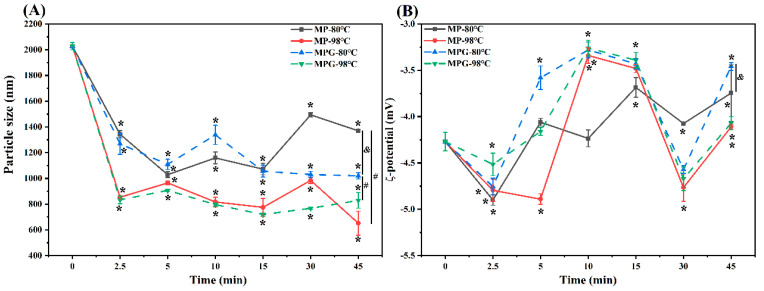
Changes in particle size (**A**) and ζ-potential (**B**) of myofibrillar protein and glucose (MPG) and myofibrillar protein (MP) under different heating conditions (80 °C and 98 °C, 0–45 min). Error bars indicate standard deviation (n = 3). Samples with * *p* < 0.05 indicate significant differences at different heating times (compared with 0 min). Samples with ^#^ *p* < 0.05 indicate significant differences between 80 °C and 98 °C. Samples with ^&^ *p* < 0.05 indicate significant differences between MPG and MP.

The particle size of MP and MPG under different heating conditions is shown in Figure 8A. During the whole heating process, the particle size of MPG and MP heated at 98 °C was much smaller than that at 80 °C. The particle size of MPG heated at these two heating temperatures rapidly decreased with heating time. For MP, at these two temperatures, except for at 30 min, the particle size decreased significantly with heating time, and the decrease of particle size in MP might be attributed to the reduction of MP clusters and more uniform dispersion of MP in solution after heat treatment [27]. In addition, the increase of particle size in MP at 30 min might be related to protein aggregation [45]; the mechanisms behind this phenomenon still need to be further investigated. It is noteworthy that, when heated at 80 °C for 15 min to 45 min and 98 °C for 2.5 min to 30 min, the particle size of MPG was significantly smaller than MP. Generally, glycation modification of protein resulted in the increase of its particle size [46]. However, the conclusions of the present study were inconsistent with empirical results. This was most likely due to the fact that, during heating, the glucose unbound to myofibrillar protein underwent auto-oxidation and generated free radicals [47], that caused the aggregation formed by glucose and myofibrillar protein to shrink in size [45].

The ζ-potential can reflect the surface charge state of particles in a dispersion system which affects the surface electrical charge [48]. As shown in Figure 8B, the changes of negative charge in MPG did not have an obvious trend with heating time. However, it was noted that the negative charge of MPG during the whole heating process at 80 °C was much less than that of MP, except at 30 min of heating. At 98 °C, the negative charge of MPG within 10 min of heating was less than that of MP, but no significant differences between them were observed with further heating. Vate & Benjakul (2016) [49] reported that protein aggregation induced by oxidized tannic acid plausibly masked the charged amino acids present in natural actomyosin, resulting in a less negative charge on the protein surface. Thus, we hypothesize that fish myofibrillar protein heated in the presence of glucose promoted protein aggregation (Figure 5B) to some degree, masking the charged amino acids in myofibrillar proteins.

### 3.8. Free Amino Content

**Figure 9:**
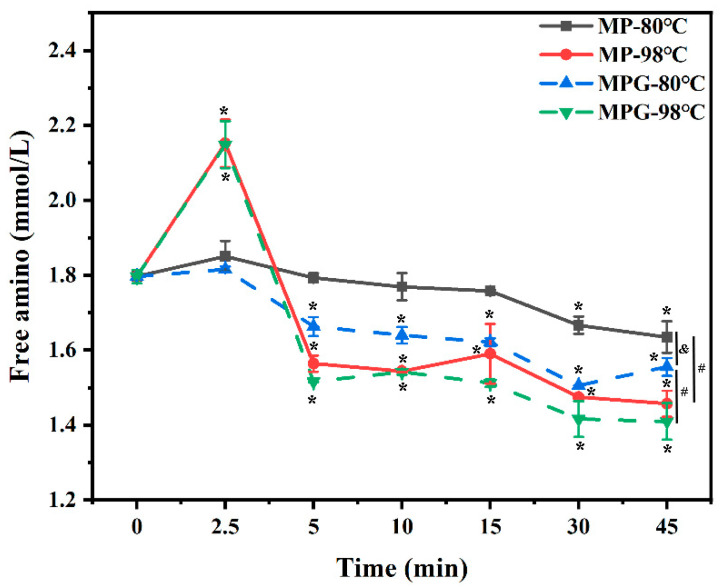
Changes in free amino content of myofibrillar protein and glucose (MPG) and myofibrillar protein (MP) under different heating conditions (80 °C and 98 °C, 0–45 min). Error bars indicate standard deviation (n = 3). Samples with * *p* < 0.05 indicate significant differences at different heating times (compared with 0 min). Samples with ^#^ *p* < 0.05 indicate significant differences between 80 °C and 98 °C. Samples with ^&^ *p* < 0.05 indicate significant differences between MPG and MP.

In a glycation reaction, the free amino content of protein and the reducing end of the sugar can form a Schiff base, and this causes the depletion of the available amino groups of proteins [50]. At the same heating temperature, the loss of free amino content from 5 min to 45 min of heating was greater in MPG when compared to MP (Figure 9). At both temperatures, the free amino content of MP and MPG rapidly increased within the first 2.5 min, and then decreased with further heating. Interestingly, the free amino content of MPG at 98 °C for 2.5 min of heating was 18.13% higher than that at 80 °C. The higher free amino content of fish myofibrillar proteins and glucose with higher temperatures during the initial heating stage could be a consequence of the intensity of heating treatments, which enhanced the denaturation of protein and caused an increase in free amino content [51]. Moreover, regardless of heating temperature, the increase of free amino content of fish myofibrillar protein heated with glucose during the initial heating stage might be due to the expansion of protein caused by the thermal treatment, thus exposing more free amino acid [13].

### 3.9. Correlation Analysis

**Figure 10:**
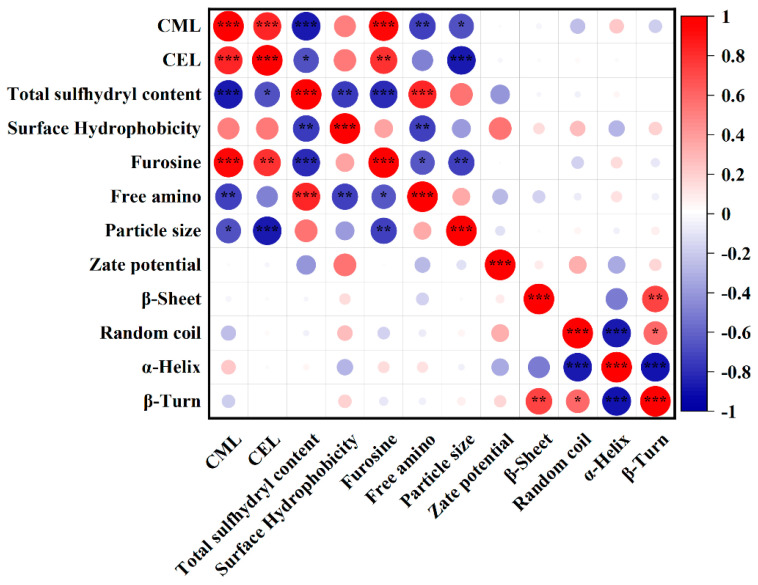
Correlation analysis of all parameters measured in this research of myofibrillar protein and glucose (MPG) under different heating conditions (80 °C and 98 °C, 0–45 min). The asterisk (*) denotes significant difference. *: *p* < 0.05; **: *p* < 0.01; ***: *p* < 0.001.

In order to further explore the effects of protein structural changes on formation of AGEs from fish myofibrillar proteins and heated with glucose, correlations between specific AGEs (CEL, CML), and the corresponding structural properties of myofibrillar proteins were analyzed by Pearson’s correlation analysis (Figure 10). The formation of CEL and CML were markedly negatively correlated with total sulfhydryl content (r = −0.68, *p* < 0.05 and r = −0.86, *p* < 0.001, respectively), but positively correlated with surface hydrophobicity content (*p* > 0.05); Furosine content was significantly correlated with CEL and CML levels (r = 0.78, *p* < 0.01 and r = 0.92, *p* < 0.001, respectively); however, there was a weak correlation between CEL and CML levels and α-Helix, β-Sheet, random coil and β-Turn. In addition, free amino content was significantly negatively correlated with CML level (r = −0.74, *p* < 0.01), but weakly negatively correlated with CEL level (r = −0.49, *p* > 0.05). Interestingly, particle size was significantly negatively correlated with CEL and CML levels (r = −0.87, *p* < 0.001 and r = −0.67, *p* < 0.05, respectively), suggesting that the formation of CML and CEL were greatly affected by the decrease of particle size during thermal treatments. The reason for this result is still not clear. Zhu et al. (2021) [45] found that, when myofibrillar proteins and glucosamine were exposed to protein peroxyl radicals (ROO⋅) derived from linoleic acid during heating, CML and CEL levels were significantly negatively correlated with particle size, which was related to the decomposition of myofibrillar proteins aggregation.

Currently, there are few studies about the obvious correlation between the total sulfhydryl content of heated food proteins and the formation of CEL and CML. However, our results showed that high CEL and CML levels were closely related to low total sulfhydryl content. Zhu et al. (2020) [1] reported that formation of a strong covalent bond between the disulfide bonds of myosin could promote the formation of CEL and CML. We hypothesized that the heating of fish myofibrillar proteins with glucose led to the aggregation of proteins via disulfide bonds, which could promote the formation of CEL and CML. Furthermore, the formation of CEL and CML levels was related to the increase of surface hydrophobicity content, but the mechanism behind this phenomenon is not clear. We speculate that the fish myofibrillar protein conformations disrupted and exposed more surface hydrophobicity groups during thermal treatments [13,29], which was related to the formation of AGEs.

**Figure 11:**
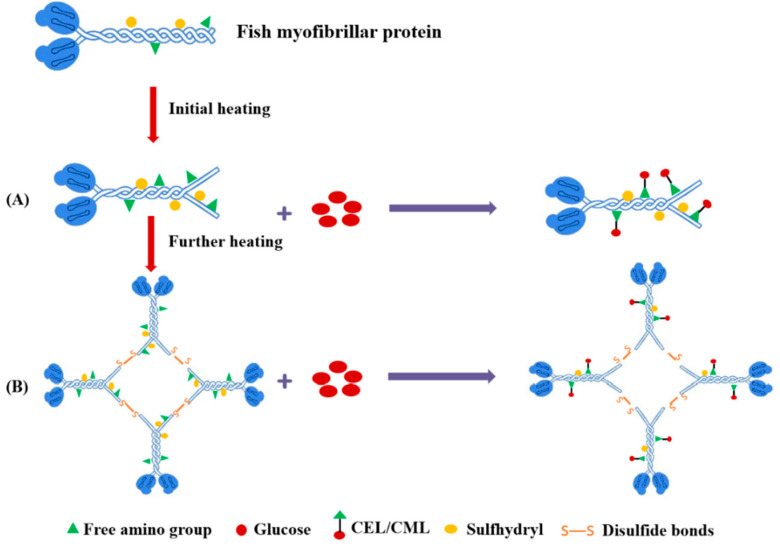
Possible mechanism for the effect of protein structure changes induced by the extent of thermal treatments on the formation of AGEs. (**A**) Initial heating. (**B**) Further heating.

The findings of the present study could mean that the formation of AGEs in fish myofibrillar protein might be affected by the protein structure changes during heat treatment, including the decrease of protein size and total sulfhydryl content and the increase of surface hydrophobicity content, which could be related to protein unfolding and aggregation. Some previous studies have also shown that the protein unfolding and aggregation induced by heat treatment might regulate the glycation process of food protein, such as bovine serum albumin, β-lactoglobulin [13] and pork myofibrillar protein [16]. Based on the results and correlation analysis presented above, a possible mechanism of CEL and CML formation in a heated fish myofibrillar protein and glucose model system was proposed (Figure 11). This formation mechanism includes the following two possible mechanisms: During the initial heating stage, fish myofibrils heated with glucose were unfolded and exposed free amino, sulfhydryl groups and surface hydrophobicity groups (Figure 11A), which were related to the up-regulation of glycation sites [13], resulting in the rapid increase of CEL and CML levels. As the extent of thermal treatments increased, fish myofibrillar proteins in the presence of glucose formed polymerization through disulfide bonds, which buried the free amino and sulfhydryl groups (Figure 11B). At this point, the aggregation of fish myofibrillar proteins caused some glycation sites to be hidden, which slowed down the formation of CEL and CML [13,14]. Based on the results presented above, it was summarized that with the increase of the extent of thermal treatments, the predominant influence factor of heating on the fish myofibrillar protein and glucose system gradually transferred from unfolding, which promoted the formation of CEL and CML, to aggregation, which slowed down the formation of CEL and CML.

## 4. Conclusions

In this study, the effects of changes in protein structure of a fish myofibrillar protein and glucose model system during thermal treatment on the formation of corresponding AGEs were investigated. It was found that the decrease of particle size, free amino content and total sulfhydryl content and the increase of hydrophobicity in myofibrillar protein and glucose during thermal treatment had a positive influence on the formation of CEL and CML. Furthermore, the correlation analysis showed that the CEL and CML levels were less affected by the protein secondary structure based on FTIR analysis. The underlying mechanism behind these results indicates that the formation of CEL and CML in fish myofibrillar protein heated with glucose was affected by its unfolding and aggregation, depending on the extent of thermal treatments. These findings help to enhance understanding of the relationship between the formation of AGEs and structure changes of fish protein during thermal processing methods, and provide some valuable references and guidelines for revealing the mechanism of AGEs formation in fish products.

## Data Availability

Date are contained within the article.
